# Parents and nurses balancing parent-infant closeness and separation: a qualitative study of NICU nurses’ perceptions

**DOI:** 10.1186/s12887-016-0663-1

**Published:** 2016-08-20

**Authors:** Nancy Feeley, Christine Genest, Hannakaisa Niela-Vilén, Lyne Charbonneau, Anna Axelin

**Affiliations:** 1 Ingram School of Nursing, McGill University, Montreal, Canada & Senior Researcher, Jewish General Hospital Centre for Nursing Research & Lady Davis Institute, Jewish General Hospital, Room H 301.1, 5790 Cote des Neiges Rd, Montreal, Quebec H3S 1Y9 , Canada; 2 Department of Nursing Science, University of Turku, Lemminkäisenkatu 1, 20014 Turku, Finland

**Keywords:** NICU, Parents, Closeness, Separation, Visitation, Nurses, Qualitative

## Abstract

**Background:**

When a newborn requires neonatal intensive care unit (NICU) hospitalization, parent and infant experience an unusual often prolonged separation. This critical care environment poses challenges to parent-infant closeness. Parents desire physical contact and holding and touching are particularly important. Evidence shows that visitation, holding, talking, and skin to skin contact are associated with better outcomes for infants and parents during hospitalization and beyond. Thus, it would be important to understand closeness in this context. The purpose of this study was to explore from nurses’ perspective, what do parents and nurses do to promote parent-infant closeness or provoke separation.

**Methods:**

Qualitative methods were utilized to attain an understanding of closeness and separation. Following ethics approval, purposive sampling was used to recruit nurses with varying experience working different shifts in NICUs in two countries. Nurses were loaned a smartphone over one work shift to record their thoughts and perceptions of events that occurred or experiences they had that they considered to be closeness or separation between parents and their hospitalized infant. Sample size was determined by saturation (18 Canada, 19 Finland). Audio recordings were subjected to inductive thematic analysis. Team meetings were held to discuss emerging codes, refine categories, and confirm these reflected data from both sites. One overarching theme was elaborated.

**Results:**

Balancing closeness and separation was the major theme. Both parents and nurses engaged in actions to optimize closeness. They sought closeness by acting autonomously in infant caregiving, assuming decision-making for their infant, seeking information or skills, and establishing a connection in the face of separation. Parents balanced their desire for closeness with other competing demands, such as their own needs. Nurses balanced infant care needs and ability to handle stimulation with the need for closeness with parents. Nurses undertook varied actions to facilitate closeness. Parent, infant and NICU-related factors influenced closeness. Consequences, both positive and negative, arose for parents, infants, and nurses.

**Conclusion:**

Findings point to actions that nurses undertake to promote closeness and help parents cope with separation including: promoting parent decision-making, organizing care to facilitate closeness, and supporting parent caregiving.

## Background

Immediately after birth the natural environment for a newborn is to be close to their caregiver [1, 2]. Physical closeness is defined as parent and infant being spatially close, while emotional closeness refers to feelings of an emotional connection to the infant such as feelings of love, warmth and affection [3]. Evidence suggests that after birth, physical closeness between parent and newborn may contribute to the development of attachment, and both parent and infant play a role in this process [4]. Parent behavior can contribute to differences in infant development, and parent exposure to infant cues and somatosensory stimulation can enhance parenting behavior and care [5].

Current understanding of the neurobiology of caregiving indicates that parents’ neurological system becomes interconnected with the newborn’s immature nervous system resulting in symbiotic regulation [6]. In this feedback system, the parent supports the biological and behavioral needs of the newborn, conversely newborn behaviors precipitate physiological processes that establish the parent-infant relationship and also stimulate neurological systems that support parental well-being. Recent evidence shows that both the brain and body of mothers undergo changes to support the development and maintenance of caregiving behaviors [7]. Imaging studies of mothers and fathers reveal that specific regions of the brain, hypothesized to be involved in parenting behavior, are activated in response to exposure to infant-related auditory or visual stimuli such as cries [7–10]. Moreover, structural changes have been observed in specific regions of the brain among mothers [11] and fathers [8] in the early postpartum. These changes may require exposure to the infant [11], and the effects of prolonged separation at this time on these neurobiological processes in parents are not known.

When a newborn requires hospitalization in a neonatal intensive care unit (NICU), parent and infant experience an unusual separation that may last weeks or months. This critical care environment poses many challenges to parent-infant closeness. NICU parents desire physical contact [12, 13], and holding and touching are particularly important and rewarding [14–17]. Mounting evidence, outlined below, shows that various forms of contact, such as visitation, holding, parent talk, and skin to skin contact, are associated with better outcomes for infants and parents during hospitalization and beyond. Greater visitation and infant holding is associated with better infant neurobehavioral development at discharge [18]. School-age NICU graduates whose mothers visited daily had fewer behavioral and emotional problems compared to their peers who had fewer visits [19].

NICU infants receiving added exposure to recordings of their mothers’ voice show lower heart rate [20], better feeding outcomes [21, 22], greater auditory cortex growth [23], better visual attention and neurofunction [24], higher Griffiths Development Quotient scores and earlier use of two word sentences [25]. Infants of mothers who spoke and sang to them daily had higher oxygen saturation levels and fewer negative critical events [26]. Furthermore, exposure to greater parent talk during hospitalization is associated with greater infant vocalizations at discharge [27], and better language and cognitive development in toddlerhood [28].

Skin-to-skin care, in which an infant lies on a parent’s bare chest, is an important form of physical closeness now promoted in NICU care, and is associated with benefits for infants, parents and their relationship. Skin-to-skin contact accelerates the development of infant’s sleep structure [29] and brain maturation [30], and lowers stress reactivity [31]. It is associated with decreased mortality, lower risk of sepsis and readmission to hospital [32]. Other benefits include shorter NICU stay, longer duration of breastfeeding, decreased parental cortisol, improved parent well-being and decreased anxiety, optimal parent-infant interaction, improved maternal attachment behavior and greater parental competence after discharge [33–41].

Policies concerning visitation and overnight stays, as well as actual visitation time and skin to skin care vary widely across units and countries [18, 42–44]. Unit policies, and the behavior and attitudes of NICU nurses play a role in parent presence and involvement. Nurses can act as gate-keepers controlling parents’ access to their infant [16, 45]. Some feel the need to seek permission from nurses to handle their infant, and their relationship with nurses influences their ability to enact their parental role [15, 46, 47]. Nurses may discourage parents from handling their infant when they consider this detrimental to infant well-being [46], and believe that skin to skin contact limits their access to infants [48]. When parents are confident and actively participate in daily infant care, nurses can feel less involved and less knowledgeable, and thus superfluous [49]. In contrast, nurses also foster parent involvement by teaching them how to care for the infant, coaching and demonstrating care, encouraging and supporting parents, enabling parent presence and providing information [50–52]. Parents perceive that nurses’ caring attitude and open communication are necessary preconditions for parent-infant interaction and bonding [47, 53]. Moreover, greater parent involvement in care and decision-making is seen by nurses as beneficial for them as well, leading to more meaningful work and improved work satisfaction [54].

Given the growing evidence that supports the importance of various forms of parent-infant proximity for parents, parenting and infant well-being, and the challenges of supporting closeness in the NICU; it would be important to further our understanding of closeness in this context. To our knowledge, no previous studies have described the nature of closeness and separation events between parents and infants during an NICU hospitalization, and what gives rise to such events in this environment. What nurses do to promote closeness or provoke separation has not been explicitly explored, and it would be important to understand their perspective to promote parent-infant closeness in practice. Thus, the questions guiding this inquiry were: What do NICU nurses consider to be closeness or separation events in the NICU? What do nurses perceive that parents do to be close to their infant? What do nurses think they do to promote closeness or cause separation? Qualitative methods were utilized to attain an understanding of closeness from the perspective of nurses in two countries.

In addition, to minimize recall bias a new innovative smartphone application was employed to collect rich data. The mobile phone application program HAPPY “Handy Application to Promote Preterm infant happY-life” was developed as a tool to collect data from staff and parents of newborns by one of the study investigators. The application allows study participants to easily record their thoughts soon after they have experience a critical incident, in this case closeness or separation between parent and infant.

## Methods

### Settings and participants

Data were collected from nurses working in two level III NICUs with different architecture in two countries. The first site was a unit in Turku Finland with 18 beds and 600 admissions per year including 50 to 70 infants weighing less than 1500 g at birth. The unit has ten single family rooms with sleeping accommodations for both parents at their infant’s bedside, and three pods of two to four beds. The second site was a 34-bed open ward unit of 400 square-meters in Montreal Canada with 650 admissions per year, including approximately 115 infants born less than 1500 g. Parents are able to be present during medical rounds and cellular phone use by staff and parents are permitted in both units. At both units, both parents are able to stay overnight: in Turku in the single family rooms on cots at their infant’s bedside and in Montreal in a parent room adjacent to the open ward unit (not at their infant’s bedside) where only one parent could stay overnight.

The study protocol was approved by the research ethics committee at each site prior to data collection. Written informed consent was obtained from each participant, and they were assured that participation was voluntary. Particular attention was paid to assuring the privacy of potential families or other staff described in the nurses’ recordings. Study participants were asked not to name individuals in their recordings. If inadvertently any identifying data was included in a recording, this information was not included in the transcript.

Nurses were informed about the study at unit meetings, via an email notice sent by the unit manager, and by posting information brochures about the study on staff notice boards. Nurses interested in participating contacted the research team by phone or e-mail for further information, and if they agreed to participate, a time to meet was arranged to obtain written informed consent. Research staff also visited the unit and approached staff asking if they had heard about the study and would be interested in participating.

At each site purposive sampling was used to include nurses with varying levels of work experience and different periods of observation (i.e., day, evening, or night). At first, any nurse meeting the inclusion criteria and agreeing to participate was included. As recruitment proceeded, the research team examined the work experience and shifts worked of the participants to date, and then sought to enrol subsequent participants who would broaden the range of experience and shifts during which recordings were made. Sample size was determined by data saturation, thus data collection continued until no new categories were evident. This occurred after there were 18 participants at the Canadian site and 19 at the Finnish site.

The characteristics of participants and their work circumstances on their study day are summarized in Tables 1 and 2. All the participants were women. There were a few eligible men at the Canadian site but none agreed to take part. Nurses in Canada were 28.4 (SD = 4.7) years of age on average, and those in Finland 38.7 (SD = 11.4) years. Although the majority worked in the NICU for more than a year, 7 of 37 (18.9 %) had less than a year of experience. Nurses recorded their thoughts primarily during a day shift, however 9/37 (24.3) recorded on evenings and 4 of 37 (10.8 %) on night shifts. Although a few nurses participated while working a night shift, there made no entries made between midnight and 7:00 am primarily because parents were not present or asleep. Half of the recordings were made of infants requiring acute or intermediate care, and half step-down care.

**Table 1:** Nurse Characteristics

	Montreal *N* = 18	Turku *N* = 19
Characteristic	N (%)	N (%)
Education		
Graduate	3 (16.67 %)	0 (0.00 %)
Undergraduate	14 (77.78 %)	19 (100.00 %)
Junior College^a^	1 (5.56 %)	0 (0.00 %)
Experience in NICU		
0 to 6 months	0 (0 %)	1 (5.26 %)
6 months to 12 months	4 (22.22 %)	2 (10.53 %)
Over 12 months	14 (77.78 %)	16 (84.21 %)
Work Experience		
0 to 6 months	0 (0.00 %)	0 (0.00 %)
6 months to 12 months	4 (22.22 %)	2 (10.53 %)
Over 12 months	14 (77.78 %)	17 (89.47 %)
Employment Status		
Full-time	9 (50.00 %)	17 (89.47 %)
Part-time	9 (50.00 %)	0 (0.00 %)
Other	0 (0.00 %)	2 (10.53 %)
Shift used HAPPY		
Day	15 (83.33 %)	7 (36.84 %)
Evening	3 (16.67 %)	6 (31.58 %)
Night	0 (0.00 %)	4 (21.05 %)
12-h day shift	N/A	2 (10.53 %)

^a^Junior college is a 3 year program after high school and before university

**Table 2:** Characteristics of nurses’ practice circumstances on the day they participated in the study

	Montreal (*n* = 64^a^)	Turku (*n* = 36^b^)
	N (%)	N (%)
Location in NICU		
Acute	6 (9.38 %)	10 (27.78 %)
Intermediate	18 (28.13 %)	18 (50.00 %)
Step-down	40 (62.50 %)	8 (22.22 %)
Single family room	N/A	27 (75.00 %)
Pod	N/A	9 (25.00 %)
Equipment required^c^		
Incubator	22 (34.38 %)	6 (16.67 %)
Respirator	6 (9.38 %)	6 (16.67 %)
Oxygen	11 (17.19 %)	^d^
CPAP	2 (3.13 %)	3 (8.33 %)
Heart monitor	33 (51.56 %)	^d^
SpO2 monitor	11 (17.19 %)	31 (86.11 %)

^a^Number of infants cared for by nurses who participated. Each nurse could be assigned several infants

^b^Number of infants that the nurses recorded closeness or separation events. They might have cared for more infants

^c^Percentages do not add up to 100 as some infants required more than one type of equipment

^d^Data not collected at that site

### Design and data collection procedure

An interpretive descriptive study was conducted [55]. Nurses were asked to use the HAPPY application over the course of one work shift. Android mobile phones with the application installed were made available to them prior to, or at the beginning of, the shift chosen for data collection. Nurses completed a background information form, and a member of the research team provided verbal and written instructions on how to use the HAPPY application. They were instructed to use the application when they experienced an event they considered to be closeness or separation to record their description of what they experienced. For example, if a nurse removed a baby from skin-to-skin contact with a parent for blood sampling, after this event the nurse would open the application on the phone and classify the event as closeness or separation by clicking the appropriate button labeled ‘separation’ or ‘closeness’. After classifying the event, they would describe aloud where the events occurred, what happened, and why. If at the time of the event, the nurse was unable to record their detailed thoughts because they did not have time at that moment they could quickly open the application and insert a bookmark. When it was convenient, they could return to the bookmark to record their thoughts.

At the end of the shift or the next day, the phone was returned to the research team. A team member downloaded the audio recorded data onto a study computer, and the recording was deleted before the phone was given to a subsequent participant. Audio recordings were transcribed verbatim by research staff fluent in the language of the recording (i.e., English, Finnish or French). Transcripts were labelled with an id number, any identifying information removed, and then captured into Nvivo software for analysis [56].

### Data analysis

To describe the events recorded by participants, we used descriptive statistics to report the total number of recorded entries, average number per participant, and the percentage classified by participants as closeness or separation events. Analysis of the data was iterative and began with the first transcripts collected, and continued throughout the data collection period. Inductive thematic analysis [57], a widely used analysis strategy in the health sciences to answer questions of clinical interest, was used. Transcript entries were coded at the site where the data were collected by an investigator fluent in the language in question. First, each transcript entry was read carefully several times by an investigator, and open-coding used to assign codes to meaningful sections of data describing closeness or separation using Nvivo [55]. To ensure trustworthiness, at regular intervals during data collection and analysis meetings of investigators were held to discuss emerging codes and conceptualizations, refine categories, reach consensus on these and confirm that these reflected data from both sites. As data collection continued, codes were compared and contrasted and eventually organized into categories, and the relationship between these categories examined. Finally one overarching theme, a thread encompassing all categories, was elaborated and a dimensional matrix employed in this process [58].

Sample excerpts from the nurses’ entries are provided in the results section to illustrate the categories. These are labeled with the participant’s identification number and site (“C” for Canada and “F” for Finland). These samples will allow readers to assess the transferability of the findings to their own setting. For the purposes of this report, examples of nurse entries made in a language other than English were translated by an investigator fluent in that language.

## Results

Study participants made 220 entries with the HAPPY application, and 158 (71.8 %) were classified by them as closeness events and the remaining 62 entries (28.2 %) as separation events. Thus, they described more closeness than separation events. Of the 78 entries made on day shifts, 78.4 (Canada) and 63.8 % (Finland) were closeness events and on evening shifts 71.4 (Canada) and 70.4 % (Finland). At both sites, the average number of entries per nurse was five, with a range of zero to 18.

### Closeness and separation events

A range of events were described by nurse as closeness events (Table 3). Nurses utilized three criteria to determine if an event was closeness or separation. The duration of visitation or presence played a role in nurses’ perceptions. If parents appeared briefly at the infant’s bedside and departed soon afterwards, nurses considered this to be separation. Conversely, spending a great deal of time at the bedside was considered an indication of closeness.

**Table 3:** Closeness and Separation Events Identified by NICU Nurses

Closeness events	Examples
Attentive presence at infant’s bedside	Being present and engaged with the infant including admiring and observing
Physical contact	Touching, hand holding or cuddling the infant while in cot or incubator, holding in parent’s arms and skin to skin contact
Events that result in increased physical proximity between infant and parent	Transfer of the infant to the ward where the mother was hospitalized or to an NICU closer to home, infant home on day pass or discharge home
Parent-infant interaction	Eye contact, talking, singing, reading, story-telling, and reacting to the infant’s cues
Typical infant caregiving and NICU care	Diapering, bathing, taking temperature, comforting a distressed infant during normative (i.e., a bath) or non-normative events (i.e., heel stick or I.V. line insertion), and feeding or participation in providing nutrition to the infant (e.g.: pumping breast milk, bottle, breast or gavage feeding)
Exchange of information between parents and NICU staff	Information about infant’s condition, care, or behavior while parents present in the unit or elsewhere (i.e., home)
Being together “as a family” with the infant	Intimate moments where family members were assembled together and visibly enjoying the experience and one another (whether mother and/or father were present, with or without siblings)
Separation events	Examples
Not physically present and no other form of contact with the infant or staff	Parents do not visit the unit or call to ask about the infant’s condition
Physically present but not engaged with infant	Parents talking together at the bedside without interest in or involvement with the infant
Departures from the bedside	Leaving to eat or sleep or care for siblings, going home or to maternity ward for the mother’s own care
Transitions from physical contact	Need to return the infant to the incubator or end skin to skin contact for any number of reasons including procedures or monitor alarm
Declining the nurse’s offer to have physical contact with the infant or provide care	Despite encouragement, the parent could not engage with infant due to own stress or discomfort

The quality of parent’s presence was also used to decide whether an event was separation or closeness. When a parent was physically present but not connected or involved with the infant, nurses considered such events to be separation. For example, one nurse described this separation event: *“The mother and father are upset about naming the baby. … Instead of being close with their baby, they’re outside of the unit now for half an hour arguing on what they’re going to name the baby.* … *(N13-C).*

Lastly, the comfort level of both parent and infant during events was another element taken into account. During a feeding for example if parent and infant were comfortable, and the infant was feeding well, this was considered closeness. If the feeding was difficult for parent or infant, then this viewed as a separation event.

In many instances where nurses described separation events, they noted parent behaviors that suggested that the parent was experiencing difficulty with the separation. Hesitation, signs of emotional distress, and repeated looking back at the infant were behaviors commonly noted.

### Balancing closeness and separation

**Fig. 1:**
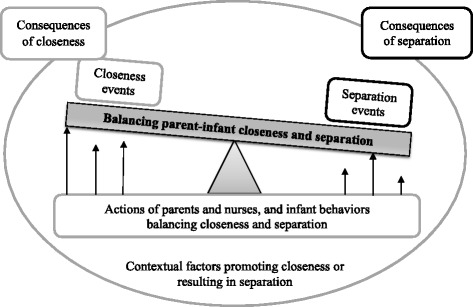
The overarching theme “balancing parent-infant closeness and separation” and the related concepts

Balancing parent-infant closeness and separation was the major theme or thread encompassing all the categories (Fig. 1). Closeness and separation events took place between parents and infants and parents and nurses could exert some control over these events. Both parents and nurses engaged in actions or efforts to optimize or tip the balance towards closeness despite the overall context of separation. Parents needed to balance their desire for closeness with their hospitalized infant with other competing demands on their time, including the needs of their spouse and other children, as well as their own personal needs for nourishment and rest. When parents confronted a separation event, they often engaged in actions to minimize separation and promote closeness. One nurse described this instance of a parent undertaking such action:

*This morning … there was a father who came in whose baby is going to be transferred to another hospital. He was not able to stay for very long, however he put a little music box next to his baby to provide comfort to his baby. I believe this was an intervention that the father did that allowed him to feel close to his baby despite only being able to be there for about ten minutes as he has a busy day ahead of him (N01-C).*

Nurses also engaged in varied actions to facilitate parent-infant closeness and minimize separation. They sought to balance the need for parent-infant closeness with the infant’s care needs and ability to handle stimulation. Infants also played a role in this balancing process, albeit a lesser one, through their behaviors and responses. A number of parent, infant and NICU-related factors influenced parents’ and nurses’ efforts to balance closeness and separation. Lastly, there were consequences, both positive and negative, that arose from closeness and separation events for parents, infants, as well as nurses.

#### Actions of parents and nurses, and infant behaviors that balance closeness and separation

As noted earlier, both parents and nurses actively engaged in behaviors that balanced closeness and separation. A myriad of parent actions were considered by nurses to promote closeness. Parents sought to balance closeness by seeking physical proximity to their infant. They did so by being present at the bedside and engaging in whatever form of physical contact was possible with their hospitalized infant at the time.

The most frequently described parental action was active involvement in infant care. When parents cared for their infant independently nurses viewed this as tipping the balance in favor of closeness. Acting autonomously in infant caregiving and being comfortable doing so is exemplified by this example: “*The father of a set of triplets arrived into the step-down unit just now. … He was not accompanied by his wife… He immediately went and started doing the care for one of his sons. I could see that he seemed very comfortable and he seemed to have a look of … pride in his eyes that he was able to give the bottle to his baby” (N01-C).*

Assuming decision-making about infant care or treatment was another parental action that nurses considered promoted closeness. In a similar vein, parents efforts to direct their infant’s care by requesting that staff perform certain caregiving activities or perform these in a particular way, or limiting staff involvement in caregiving was a related category of actions. In one instance a mother called the unit and requested that the nurse not perform any infant care that morning so that she could do so when she arrived.

Parents seeking information about their infant’s medical status or care was considered to help attain closeness. One nurse remarked*: “To me … the parents calling to ask ‘How is my baby doing, what is the weight this morning, did she eat well’… it is a sign of interest in the baby and a way to be close” (N04-C).* Verbal statements or other actions undertaken that reflected parent’s interest in the infant or desire to be involved in care was another related form of action noted. In one situation a mother asked the nurse if she could provide care for her infant that she had not previously performed. Similarly seeking feedback, advice about or acquiring skills in infant care were also viewed to promote closeness.

When separation was required, parents engaged in familiar goodbye rituals such as bidding farewell, kissing, and promising to come back as soon as possible to cope with these events. Parents also undertook actions to establish a connection during a separation. They did so by leaving objects such as a music box or toy with their infant, calling the unit and asking the nurse to tell the baby that his parent loves him, or taking pictures of the infant to take home.

In this complex care environment, strategies that nurses employed to balance closeness and separation were also varied. Supporting parents’ autonomy in infant care was a key strategy. Nurses promoted parent autonomy by teaching, demonstrating care or procedures, engaging with parents to jointly provide care or by expressing their availability to be of assistance if needed as the parent performed caregiving. Only the Finnish nurses described helping parents learn about infant behavior. They shared their observations of infant behaviors or preferences with parents, or engaged parents in making observations of infant behaviors.

Promoting and respecting parent’s role as the decision-maker for their infant was a way to balance closeness and separation. For example, one nurse had a discussion with parents about when to do a care procedure. In a similar vein, providing and or facilitating access to information about the infant’s condition or care was considered to promote closeness. Nurses also advocated on parents’ behalf with other staff: “*The baby was very hungry. I discussed with the mother about her baby’s fasting and promised to ask the doctor if the baby can have some breast milk although the operation was done just two days ago”. (N16-F)*

Nurses encouraged parents to be present and have physical contact with their infant (e.g., encouraging touch or visual contact when that was all that was possible, or breastfeeding and skin to skin contact). Controlling aspects of the NICU environment, such as providing privacy, reducing noise during parent-infant contact; and structuring care to support parent involvement (e.g., setting equipment up to facilitate skin to skin contact) was another category of nursing actions. “*The baby is intubated. The father lifts the baby very calmly back into the incubator from skin to skin contact on his mother. He has evaluated that the boy is ready for the transition. The baby is awake, not asleep. The transition goes well. My role as a nurse is to move the ventilator and the tubes while the father places the boy nicely back into the incubator.” (N13-F)*

Providing emotional support was viewed as enhancing closeness. For example, nurses provided positive feedback to parents about their abilities to care for their infant or reassured them about their infant’s well-being. Lastly, nurses recognized parents’ struggles with separation events and tried to ease these events by promising to take good care of the infant while the parent was absent.

Although most of the nurses’ entries involved promoting closeness, there were situations in which their actions resulted in separation. NICU nurses sometimes decided that stimulation to the infant needed to be controlled and this provoked a separation event. In one case, the infant was sleeping and the nurse recommended that the parents not wake the infant to hold him as they wished to do. When nurses intervened to provide a required treatment, such as oxygen, this also resulted in separation. If parents requested information and the nurse was unable to provide it, this was perceived to contribute to separation. Occasions also arose where a parent sought the nurse’s assistance while caring for their infant; however the nurse was occupied and unable to respond to the request, resulting in separation.

The behavior of the infants played a role in balancing parent-infant closeness. Infant signals such as crying, fussing, facial expressions, vocalizations, and change in vital signs typically prompted a parental response. In this example the infant emitted a signal and the parent responded, resulting in a closeness event. “*The infant was in the crib while the mother was pumping her milk and the father was helping, a moment of closeness occurred when each time the baby stirred or made a sound, the father would get up from his chair and check on the infant and comfort him by patting him, offering him the pacifier and speaking to him softly to console him” (N17-C).* In contrast, infant crying while parents engaged in caregiving could provoke feelings of inadequacy, and this was viewed as a separation event.

#### Factors influencing closeness and /or separation

Embedded in nurses’ descriptions of closeness and separation events were parent, nurse, infants, and contextual factors that nurses perceived influenced the balance between closeness and separation. Parent characteristics such as lack of child care experience, distressed emotional state, and poor health status (e.g., pain, fatigue or hospitalization) tipped the balance towards separation. Worry about the infant’s medical condition or fear of the infant’s or mother’s death were examples of these. One Finnish nurse explained: *“I encouraged the father to hold the baby as she was crying in the cot, but at that point he was not able to do so because of all his worries (the mother was in the ICU). He needed support from me more than closeness with the baby” (N12-F).* In contrast, when a parent was confident in their ability to care for their infant or had previous child care experience, these attributes promoted closeness. Nurses own beliefs played a role. When they believed that parents should be the main caregivers this enabled closeness.

The infant’s physiological stability could affect closeness and separation*.* When an infant experienced an adverse event such as desaturation or bradycardia while in the parent’s care, nurses noted the parent verbally expressed or appeared to feel inadequate. Conversely when one mother who was afraid to hold her infant noted that the infant’s vital signs remained stable when she held him, she relaxed and enjoyed the physical closeness.

With respect to contextual factors, the overall atmosphere of the NICU, as well as specific aspects of it, played a role. When the environment was quiet and calm, this enabled parent-infant closeness. As one nurse stated: “*In a single family room the father is holding his boy in skin-to-skin care and the mother is sitting at the foot of the bed. The atmosphere is calm and convivial which contributes to closeness” (N13-F).* While the environment could promote closeness, it could also influence separation. Lack of privacy, equipment noise and the overall technological atmosphere were viewed as contributing to separation. Infant care requirements such as treatments or procedures, the incubator, equipment or devices were other environmental factors as illustrated in this example: *“The infant had an urinary catheter and was intubated so the parents could not get close to him” (N14-F).*

Only Finnish nurses considered that how care was organized in their hospital influenced closeness. They reported that because mothers and infants were hospitalized in separate units women were forced to leave their infant and the NICU to go to the maternity ward for their own medical care: “*The mother had to put the infant into the cot and go back to her own ward for breakfast and morning wash” (N06-F).*

When parents attended to their own personal needs for rest and nourishment this contributed to separation, as did attending to the needs of other family members. Parents had to balance other responsibilities that required their time and attention, such as other children, family members and employment. Some fathers needed to attend to the needs of their partner particularly when the mother herself was unwell after childbirth. Parents of multiples confronted particular challenges as they sought to balance closeness with more than one infant.

#### Consequences of closeness and separation

Nurses identified consequences of closeness and separation events for parents, infants and nurses. Nurses balanced these consequences when making decisions about infant care. The main dilemma for them was whose needs should be given priority; the infants’ or parents’? A Finnish nurse expounded on this challenge:

“*This skin to skin contact event made me think about whose needs should determine the timing of skin to skin contact. The infant was in deep sleep in the incubator, and this is important for development. The parent wanted to have her in skin to skin contact at that time. What is best, that the parent have her infant her close or that the infant should remain in deep sleep?” (N04-F).*

Closeness events typically gave rise to positive consequences for parent and or infant, nonetheless this was not always the case. Positive consequences for parents included improved parental mood (e.g., happy, proud), greater comfort in caring for their infant, improved engagement with the infant, better maternal milk production, and feeling useful. One nurse reported: “*I gave a bath demonstration for baby Y today and mommy felt really happy to be able to give the bath to the baby. She felt comfortable and she felt that she was useful. … It was a good thing for her to start giving baths to her baby (N07-C).* There were situations where nurses identified negative consequences of closeness for parents, such as when parents experienced extreme fatigue when they had extended closeness with the infant and did not get sufficient sleep.

Consequences of closeness for the infant were all positive, and included improved infant state (e.g., alert, satisfied, calm, falls asleep), stabilization of vital signs, and effective feeding. As one nurse explained:

…*mom was standing at the bedside while the nurse was gavaging the baby. Mom had her hand inside the incubator and she was trying to calm her baby down, placing her hands on the baby’s head and touching her baby and because of that everything went smoothly and the baby was able to receive her feed a lot more easily and was less agitated and not crying as much because mom was present (N11-C).*

Interestingly, nurses also reported consequences for themselves when they were involved in, or experienced a closeness or separation event. Situations in which closeness events slowed the nurse’s work were evident, nonetheless they made a conscious decision to support closeness despite adverse effects on their own work. Nurses also felt touched emotionally when they experienced parent-infant closeness.

In contrast to closeness events, most separation events were associated with negative consequences for parent and/or infant. Negative consequences of separation for parents included feeling out of control or useless and negative mood (e.g. anxious, sad, or guilty). One nurse reported: “*The baby did a desaturation with the feed of a bottle. This was a separation because I had to take the baby from the mom. … The mom felt a little bit useless at that moment because she couldn’t help her baby” (N07-C).*

When nurses provoked separation, guilt feelings arose. Sadness surfaced when they experienced a separation event. Learning could also be a consequence for nurses. They noted that when they will encounter a similar situation in the future, they intend to change their approach. In this example the nurse learned what needed to be avoided to prevent separation between one father and his infant: “*The baby had an I.V. line in the head. The parents came to take care of her and when the father saw the I.V. cannula he was terrified. He was forced to leave the room and only able to come back when the baby had a cap on her head. I was so disappointed for the parents because I did not know about the father’s fear and was not able to prevent this separation by putting the cap in the baby’s head in advance” (N09-F).*

## Discussion

The ultimate goal of NICU nurses is to provide a care environment that will support infant development, and physical and emotional closeness between parent and infant has important benefits for infant development. However, there are many aspects of this critical care environment that pose challenges to closeness. Thus a nursing care culture that supports closeness is imperative. We found that from nurses’ perspective, both parents and nurses engage in varied actions to balance parent-infant closeness in this overall context of separation. Our findings point to care practices that could support closeness, the well-being of parents, and parenting in the NICU; and in turn the development of infants. Implications for practice are elaborated below and summarized in Table 4.

**Table 4:** Implications for clinical practice

To optimize parent-infant closeness NICU clinicians should:	
• Support parents to be able to care for their infant autonomously • Provide emotional support to parents • Optimize parents physical proximity to their infant (e.g., skin to skin contact, holding) • Engage in shared decision-making with parents to arrive at care decisions that are optimal for infants and their families, and acknowledge parents’ key role • Be aware of the difficulty of separation events for parents, and help them develop strategies to cope with these events such as goodbye rituals when they need to leave the bedside • When clinical staff must separate parent and infant for care requirements, they should be cognizant of parent’s responses and acknowledge the difficulty of these events for parents.	

Nurses identified numerous closeness and separation events, and used the duration and quality of engagement of the parent, as well as the comfort of both partners during the event to determine if they considered that event to be closeness or separation. They considered that physical closeness could facilitate emotional closeness and vice versa. However there could also be a disconnection: parents could be physically close but emotionally detached, or physically distant but emotionally connected.

Interestingly our findings coincide with the key attributes of parent-nurse partnership identified in a concept analysis of family-centered pediatric care; namely parent autonomy and control, shared responsibility for decision-making and caregiving, and negotiation about care [59]. This suggests that family-centered care could facilitate NICU parent-infant closeness. From Canadian and Finnish nurses’ perspective, fundamental actions that parents engaged in to promote and balance closeness were acting autonomously and playing an active role in decision-making. On the other hand, nurses balanced closeness by supporting parents to act autonomously in caregiving. For decades, studies have consistently documented the stress NICU parents experience due to restriction of their parental role and its adverse effects on the parent-infant relationship [16, 46]. In contrast, when parents care for their newborn as they expected to do so if the infant had been born at term this promotes a sense of normality, fosters closeness [16], and promotes parental well-being and competence [15, 46, 51].

We found that information-seeking and learning how to care for their infant were parent actions enhancing closeness. Conversely, by providing parents with information, supporting their access to information from others sources, and teaching and coaching parents in developing care knowledge and skills, nurses facilitated closeness. Moreover, when parents played an active role in decision-making and directed infant care this too promoted closeness. Previous studies highlight the significance of information [60] and the challenges parents encounter negotiating with nurses, challenging practices, and insisting that things be done as they prefer [46]. De Rouck and Leys [61] argue there should be “information exchange” between parents and NICU staff: staff provide technical information while parents provide personal information (i.e., their own preferences and those of their infant) to arrive as care decisions that are optimal for infants and their families. This process has also been referred to as shared decision-making [62]. For this to happen, staff must relinquish control, truly exchange information and collaborate in decision-making with parents.

Close Collaboration with Parents and Family Integrated Care (FICare) are new models of NICU care that may benefit families and staff. The Close Collaboration with Parents program aims to strengthen the implementation of FCC by training the entire nursing staff of a unit to conduct joint observations of infants’ behavior with parents. This individualized information is used for planning infant care collaboratively during hospitalization and the transition to home [63]. Finnish nurses trained in the Close Collaboration program regard greater parent involvement in infant care and decision-making as beneficial for both families and themselves [54]. Closer relationships with parents translated into more meaningful work and improved work satisfaction.

The FICare model involves parents acting as primary caregiver, taking part in medical rounds and collaborating with professionals in developing the care plan [64]. Nurses and other staff play a secondary role supporting parents, thereby enhancing both parent autonomy and infant well-being. The effects of this model are now being investigated in a clinical trial [65, 66]. Although these models hold great promise to minimize the adverse effects of a NICU hospitalization for parents and infants, it is imperative to consider that not all parents will desire this level of involvement and some may require more time and support than others to assume such roles [17]. It seems likely that parent involvement should best be tailored to parent’s personal preferences [59]. For example in this study, nurses reported separation events when parents declined their offer of contact with their infant or an opportunity to provide care.

From nurses’ perspective, providing emotional support to parents was perceived as fostering closeness. Further, parents’ psychological and physical well-being was a factor in their ability to be close to their infant and vice-versa, closeness improved parents’ mood and well-being. NICU staff need to provide support and resources that parents may require to improve cope with any emotional distress they may experience so they are able to be physically and emotionally close with their infant. Recently the National Perinatal Association in the United States developed standards for the psychosocial care of NICU parents [67]. They recommended that all parents have a meeting with a mental health professional assigned to the unit within 3 days of their infant’s admission, and that all parents should be screened for emotional distress (i.e., depression and post-traumatic stress) [68]. A referral system needs to be in place to provide care to those parents who may require this.

Nurses have a vital role to play in controlling noise and providing privacy to support closeness. It is known that noise and lack of privacy are environmental factors that affect parents’ NICU experience [69, 70]. Our study extends this knowledge by highlighting the role that these environmental features play in parent-infant closeness. Staff need to be cognizant of these factors and minimize their impact on parents. Moreover, the extent to which the architectural design supports closeness needs to be addressed when planning new units [71]. Attention needs to be paid to unit décor as it should be more home-like than institutional [50, 72].

Our findings indicated that seeking physical proximity was a key parental action to balance closeness. Parents want to be physically present to form a relationship with their infant. Although telephone calls or other innovative forms of remote communication now being explored (e.g., web-cam viewing of the infant from home) may help parents cope with NICU hospitalization, evidence suggests that physical presence and contact remains preferable for parents [73]. This is not surprising given current knowledge of the biological mechanisms underlying parenting and attachment. If physical contact is so critical, early in the hospitalization nurses need to assess parent visitation and help parents manage barriers to their presence [43].

Nurses strive to balance the need for parent-infant closeness with the needs of the infant and their ability to handle stimulation. Based on principles of developmental care, staff may discourage parents from handling their infant when they consider this might be detrimental [46]. As seen in this study, they may remove infants from their parents embrace when an adverse event occurs. If this is necessary, nurses should do so in a way that minimizes deflation of parental competence. Also they should contemplate whether the event could be managed without removing the infant. The natural environment for an infant is close to their caregiver and they could recover from adverse events if nurse can learn to work in this new context and support parents as well. Lastly, nurses should do a thorough assessment of the infant’s ability to manage handling, and prevent adverse events from occurring.

Separation events were typically characterized as difficult for parents as evidenced by the distress nurses witnessed. A Swedish study described variability in parents responses to departure based on their comfort with the staff, and the stability of the infant’s medical condition [69]. Our data also showed that parents engaged in actions to maintain a connection in the face of separation. Nurses must be cognizant of the difficulty of departures for parents. Together with parents, they can develop an approach to departures that might ease parent’s distress. Goodbye rituals, transitional objects and leaving when the infant is asleep seemed to help parents cope. In addition, staff should appreciate the value of telephone contact to parents when they cannot be physically present. Transitions from close contact, such as the end of skin to skin care, can also be managed with similar attentiveness to parent’s feelings.

### Limitations and directions for future research

One limitation of our study is that participants are likely to be nurses who are interested in promoting closeness and minimizing separation. Nurses described guilty feelings when reporting that they had caused a separation event, as they may perceive that closeness is more desirable than separation. This may explain why there were fewer separation events reported compared to closeness events, and why there were fewer examples of nurses’ actions resulting in separation events. As well, nurses may have changed their behaviors during the study period as they may also be more aware of their actions and how this impacted on parents’ relationship with their infant. In addition, this study captured nurse’s perspectives on closeness and their perspective is likely to be different from that of parents. A study using the same methodology is underway at both study sites to explore parents’ perceptions. Having data from the perspectives of both groups will provide an in-depth understanding of parent-infant closeness during NICU hospitalization. A strength of our study is that the findings are based on the perceptions of nurses’ from two different countries practising in units with different types of design: one single family rooms and the other an open ward.

## Conclusion

From nurses’ perspective, both parents and nurses engage in actions to optimize closeness. Nurses considered that parent, infant and NICU-related factors influence closeness. Consequences of closeness and separation events arise for parents, infants, and nurses. It will be critical to understand if the strategies employed by nurses are also perceived by parents to promote closeness and minimize separation, as their point of view is essential.

## Abbreviations

HAPPY, handy application to promote preterm infants happy life; NICU, neonatal intensive care unit
